# A qualitative study of primary care professionals’ views of case finding for depression in patients with diabetes or coronary heart disease in the UK

**DOI:** 10.1186/1471-2296-14-46

**Published:** 2013-04-04

**Authors:** Margaret Maxwell, Fiona Harris, Carina Hibberd, Eddie Donaghy, Rebekah Pratt, Chris Williams, Jill Morrison, Jennifer Gibb, Philip Watson, Chris Burton

**Affiliations:** 1 Nursing, Midwifery and Allied Health Professions Research Unit, University of Stirling, Iris Murdoch Building, Stirling, FK9 4LA, UK; 2 Community Health Sciences, University of Edinburgh, Edinburgh, UK; 3 Department of Family Medicine and Community Health, University of Minnesota, Minnesota, USA; 4 University of Glasgow, Glasgow, UK; 5 Robert Gordon University, Aberdeen, UK

**Keywords:** Depression, Case finding, Screening, PHQ9, Diabetes, Coronary heart disease, Primary care

## Abstract

**Background:**

Routinely conducting case finding (also commonly referred to as screening) in patients with chronic illness for depression in primary care appears to have little impact. We explored the views and experiences of primary care nurses, doctors and managers to understand how the implementation of case finding/screening might impact on its effectiveness.

**Methods:**

Two complementary qualitative focus group studies of primary care professionals including nurses, doctors and managers, in five primary care practices and five Community Health Partnerships, were conducted in Scotland.

**Results:**

We identified several features of the way case finding/screening was implemented that may lead to systematic under-detection of depression. These included obstacles to incorporating case finding/screening into a clinical review consultation; a perception of replacing individualised care with mechanistic assessment, and a disconnection for nurses between management of physical and mental health. Far from being a standardised process that encouraged detection of depression, participants described case finding/screening as being conducted in a way which biased it towards negative responses, and for nurses, it was an uncomfortable task for which they lacked the necessary skills to provide immediate support to patients at the time of diagnosis.

**Conclusion:**

The introduction of case finding/screening for depression into routine chronic illness management is not straightforward. Routinized case finding/screening for depression can be implemented in ways that may be counterproductive to engagement (particularly by nurses), with the mental health needs of patients living with long term conditions. If case finding/screening or engagement with mental health problems is to be promoted, primary care nurses require more training to increase their confidence in raising and dealing with mental health issues and GPs and nurses need to work collectively to develop the relational work required to promote cognitive participation in case finding/screening.

## Background

Chronic physical illnesses, such as Diabetes Mellitus (DM) and Coronary Heart Disease (CHD) are associated with increased prevalence of depression [1-4]. Recent expert guidelines recommend systematic case finding (also commonly referred to as ‘screening’ by clinicians and often combined in the review literature) for depression in patients with DM [5,6] and CHD [7]. However, there is some evidence that case finding/screening is only effective when there are further systems in place to support on-going management of depression and the continued involvement of staff engaged in screening processes [8-11].

The UK National Health Service (NHS) introduced annual case finding for depression in patients with DM and CHD in 2006 as part of the General Practice Quality and Outcomes Framework (QOF) contract [12] using two validated screening questions ^a^[13-15]. Further assessment is provided to those giving a positive response to either question. In practice, case finding/screening is often carried out by nurses as part of chronic disease management reviews but many have little or no specific mental health training [16]. Since case finding using ‘screening questions’ is more commonly referred to as ‘screening’ by practitioners and in the review literature [7-11], we use the term ‘screening’ throughout the remainder of this article as generally reflecting the process of using the Whooley questions as well as more detailed assessment tools (such as PHQ9 or HADS) to detect depression in DM and CHD patients.

There has been little research into how such changes in depression care are implemented in everyday practice and from a theoretical perspective. One study has qualitatively explored GP and patient perspectives of the use of depression severity measures and found that GPs prefer to use their clinical judgement [17]. Another study of GP views of the routine introduction of measures of severity of depression through the QOF reported similar findings with GPs preferring to use scores to aid clinical judgement [18]. A further study also qualitatively explored primary care practitioner perspectives on the impact of the QOF and NICE guidelines on the diagnosis and management of depression, and found screening and severity assessment negatively impacted their work. Only this latter study included, and briefly reported on, nurse perceptions of screening within chronic disease management reviews [19].

Gunn et al. [20] examined the work involved in implementing improved depression care by clinicians using Normalisation Process Theory [21] to develop a conceptual framework for effective depression care. This suggested the need for coherence in terms of who is, or is not, depressed; cognitive participation by professionals using a shared set of techniques to actively engage and ‘join in’ with depression work; and collective agreement about how care is organised. Cognitive participation is the ‘relational work’ that people do to adopt and sustain new practices (or technologies or interventions) and involves: ‘initiation’ by key participants who work to drive change forwards; ‘enrolment’ of participants so they *collectively* contribute to the adoption of new practices which may require rethinking and reorganising of individual and group relationships and roles; ‘legitimation’ to ensure that those involved believe that the new practice can be part of their role and that they can make a valid contribution; and ‘activation’ to collectively define the activities and processes needed to sustain the new practices [22].

The introduction of routine case finding/screening for depression among patients with long term conditions in the UK (as incentivised within the QOF) has represented a large scale natural experiment of the introduction of guidelines which are derived from studies conducted within a research controlled environment. This offered an opportunity to explore how these have been implemented in everyday practice: how they are understood and interpreted by frontline clinicians, what level of coherence and cognitive participation in depression work exists to support screening, and how clinician and patient interactions might impact on the fidelity and outcomes of the intended intervention. A separate quantitative analysis suggested only modest effects of screening carried out by primary care practices in terms of cases detected [23].

We carried out two complementary focus group studies with primary health care professionals to explore their experiences of implementing screening for depression in chronic illness, with a particular emphasis on practice nurse experiences of depression screening in primary care.

## Methods

This paper is based on the combined results of two studies that explore similar populations and thus have complementary findings. We believe that by combining these two studies we achieve a larger sample and a wider range of views on this important topic.

### Participants and sampling

Study 1 was part of a feasibility study for a practice nurse-led self help intervention for depression in people with DM and/or CHD conducted in 2009–2010. We approached 22 general practices registered with the Scottish Primary Care Research Network as having an interest in long term conditions and five of these agreed to take part. All practices participated in the Quality and Outcomes Framework (QOF), a UK pay for performance scheme to incentivise particular clinical activities. All GPs and nurses from the five practices were invited to attend a focus group arranged at a time to accommodate maximum participation. Fourteen professionals (6 GPs and 8 Practice Nurses) from the five practices took part in four focus groups conducted on practice premises (see Table 1).

**Table 1:** Focus group participants

	**No. of participants**	**No. of GPs**	**No. of practice nurses**	**No. of long term condition /specialist nurses**	**Others (CHP managers; other service providers)**
**Study 1**					
Group 1	3	3	-	-	-
Group 2	4	-	4	-	-
Group 3	2	-	2	-	-
Group 4	5	3	2	-	-
**Total**	**14**	**6**	**8**	**0**	**0**
**Study 2**					
Group 1a CHP1	9	1	1	3	4
Group 1b CHP1	7	1	1	3	2
Group 2a CHP2	7	2	1	2	2
Group 2b CHP2	7	2	1	2	2
Group 3a CHP3	7	2	1	3	1
Group 3b CHP3	7	2	2	2	1
Group 4a CHP4	10	2	2	4	2
Group 4b CHP4	7	1	2	3	1
Group 5a CHP5	8	-	1	3	4
Group 5b CHP5	7	1	1	3	2
**Total**	**76**	**14**	**13**	**28**	**21**

Study 2 took place within a larger quality improvement project (conducted in 2008–2011) targeting the identification, assessment and care of depression in people living with long term conditions. The quality improvement project took place within five Community Health Partnerships (CHPs) ^b^ across four Health Board areas of Scotland. In each participating CHP, two volunteer practices were recruited to facilitate the associated research which aimed to guide the improvement process and assess potential impact. All practice staff (GPs, Nurses and Nurse specialists) attached to the research practices were invited by letter to a focus group alongside CHP level practitioners and managers with responsibility for improving long term condition management (as identified by local CHP management leads). Practice managers advised about the best time to conduct groups to maximise possible attendance. A total of 76 clinicians or NHS managers took part in 10 focus groups organised around the 10 ‘research practices’ (see Table 1).

The focus groups in both studies were undertaken as part of wider study aims: study 1 focus groups covered the acceptability and feasibility of conducting a nurse-led intervention for depression in patients with DM and/or CHD which included nurse screening for depression; study 2 focus groups covered how primary care (and the CHP) might better respond to the mental health needs of their patients with long term chronic illness, including processes for early detection (screening) and response. Focus groups were deliberately chosen to reflect the shared nature of chronic disease management in primary care. In both studies, focus groups were approximately 1 to 1.5 hours duration, although not all of this time was devoted to the discussion of screening. Study 1 participants were asked how able they felt to discuss mental health issues with patients; and how did they currently manage and support people with DM or CHD in relation to mental health. These questions initiated discussion and further probing around screening processes as this was an important process within the feasibility study as it was being used as a mechanism of recruitment. Study 2 asked a general question regarding the annual review (How effective do you think the current Annual Review is in addressing the mental health and mental well being of people with diabetes and/or CHD?) which initiated discussion around screening and prompted further probing of screening and subsequent management.

Due attention was paid to including practices located in both affluent and deprived areas as well as both urban and rural areas. Details of group participants from both studies are summarised in Table 1.

### Research conduct

The research team represented a broad range of disciplines, including social science (MM, FH, ED, RP, PW), clinical primary care (CB, JM, JG) and psychiatry (CW). This disciplinary mix ensured that the researchers were informed by social science theories and techniques as well as expert medical and psychiatric guidance. Focus groups were conducted using semi-structured topic guides with emerging themes informing questions for future groups. The researchers in both studies obtained signed consent to participate, to recording of the session, and to using anonymised findings in subsequent reports and publications. Trent NHS Research Ethics Committee granted ethical approval to Study 1 (REC ref 08/H0405/39) and NHS ethics exemption was confirmed by Scotland A REC for Study 2 (on the grounds that it was deemed as audit and evaluation of local quality improvement projects). Study 2 was still subject to and granted ethical approval by the University of Stirling.

### Data analysis

Focus groups in both studies were digitally recorded, transcribed and analysed for recurrent themes using a method of constant comparison [24]. The analysis was informed by an interpretive approach, based on the constructivist version of grounded theory [25] as its epistemological underpinning but conducted in a pragmatic manner (not a-theoretical and not involving theoretical sampling). Data handling was facilitated by use of NVivo 8 software. Analysis of Study 1 data was conducted by FH following discussion and agreement of key themes with MM, CH, RP and CB. Analysis of Study 2 data was conducted by ED following discussion and agreement of key themes with RP and MM. From both studies, we extracted themes relating to screening for depression in long term conditions in primary care, how this is generally conducted within practices, and experiences and views of how QOF screening addresses the mental health of people with long term conditions. The content of these broad themes (descriptive) was then consolidated into the three analytical themes. Those involved in analysis across the two studies met twice to discuss and consolidate themes and confirm analytical interpretation of findings and presentation of data. The consistency of themes and supporting data means that a collective presentation of the analysis has been possible.

## Results

Participants in both studies reported that most screening was conducted by practice or specialist (DM or CHD) nurses, therefore much of the data is focused on nurses’ experiences and concerns. We present findings in relation to three key topics: barriers to incorporating depression screening into a routine consultation; replacing a naturalistic and individualised approach to distress with mechanistic questioning; and disconnection for nurses (in terms of process, knowledge and skills) between physical and mental health.

### Barriers to incorporating screening into the routine review consultation

The method of screening that participants reported that they aimed to use was the two screening questions, then, if there was cause for concern, following this up with a further assessment tool such as the Patient Health Questionnaire (PHQ-9) [26], Hospital Anxiety and Depression Scale (HADS) [27] or Beck Depression Inventory (BDI) II [28], either during the consultation or later in accordance with guidelines. However they reported concerns about the way screening was incorporated into the consultation which suggested that not only was it difficult, but that it may bias the results. Time constraints were a particular problem:

I think the screening questions are seen as a sort of tick box exercise. Also there’s not time, you know, we have twenty minutes/half an hour, we’ve to do their feet, BP, cholesterol and right at the end it’s ‘are you depressed?’ ‘no?’, (phew!) that’s fine, next!…(Study 2, Group 1b, Specialist Diabetes Nurse)

For most nurses, the inclusion of questions on emotional health at the end of a long list of physical health priorities minimised its importance. The resultant manner in which the questions were administered discouraged patients from disclosing any problems.

You know, the evidence of mental health problems in people with chronic disease is very high, but we don’t seem to pick up as many perhaps as we should be. And I think that’s because the screening questions are just perhaps fired at people and they go, “Well fine, thanks very much … well, that’s okay then”. (Study 1, Group 1, GP).

The problem of time for the consultation and screening extended to the problem of dealing with a positive result; with concerns that the clinician might be overwhelmed by opening a ‘Pandora's box’ or ‘can of worms’. As a result, questions may be asked in a way which discouraged the patient to respond:

GP1: And when this QOF stuff came out, you know, I think we all thought ‘well it’s great identifying it, but what are we going to do with the extra 300/400 patients who identify with mild anxiety and depression?’. [....] So one way of dealing with it of course is not to deal with it…

GP2: Just ignore it.

GP1: And let’s ignore, well we ask the question, but not in a way…[participant interrupted by another] (Study 1, Group 1, GPs)

Nurses also reported concerns about a lack of services or options available if people were identified as depressed. This suggests a lack of knowledge or confidence for both GPs and Nurses concerning the availability of resources to help manage depressed patients.

Nevertheless, despite similar reservations, one GP commented that having the two questions built into annual reviews ensured that screening for depression was not forgotten: *“there’s something there about you working with a template that prompts you to do it…”* (Study 1, Group 4, GP).

### Replacing an individualised naturalistic approach with mechanistic screening

The introduction of recommended tools was reported by both nurses and GPs as replacing a more holistic discussion with patients. They described this more mechanistic process as ‘less professional’, and disrupting the normal patient/professional interaction. Nurses felt that the scripted questions required more surrounding dialogue.

‘The QOF questions are progress in tackling this issue but a lot of us don’t like using PHQ9 because we’re sitting speaking to the patient, you then print off this sheet, give it to them to fill in rather than engaging verbally … it’s really much less professional I think most of us feel, but we have to do it, so…’(Study 2, Group 3a, Specialist Nurse)

This mechanical reliance on formal measures was portrayed as superfluous to some nurse’s professional skills and instincts:

“So I think in the half hour you get a good idea of whether someone is… this is just a bad day, or whether there’s been a lot of bad days… And I think your instinct kicks in, you know?” (Study 1, Group 3, Practice Nurse).

### Disconnection between physical and mental health

Most nurses reported that their professional role, until recently, had not included mental health and while they valued the recognition of its role in wider health, they required a better understanding of mental health to more effectively introduce screening to patients.

Because if you (nurse) don’t really know why you're doing it then you're not going to be able to gauge that question properly in order to get the most accurate answer. Because you want to say to people ‘this (diabetes/CHD) can affect your mental health and your mental wellbeing’ and you want to kind of give them an explanation of why you're asking them about this, not just ‘oh I have to ask this question’… (Study 2, Group 1b, Specialist nurse)

The lack of training preceding the implementation of screening may account for some of the failure of nurses to adopt mental health awareness and promotion as part of their role and to develop appropriate skills to engage effectively with patients. Indeed one nurse reported: ‘*We’ve been floundering for a couple of years’* (Study 2, Group 3a, Specialist Nurse).

In other instances, the nurses’ own lack of confidence prevented them from challenging patients’ reluctance to seek help, thereby missing potential opportunities to intervene.

This lack of confidence in dealing with the consequences of disclosure of mental health problems by patients made nurses feel vulnerable: emphasised their lack of skills, and was considered unsatisfactory for patients who had made disclosures to then have their discussion curtailed.

It’s not like taking somebody’s blood pressure or measuring somebody’s weight. It’s like how to approach the subject and how to appropriately respond because […] let’s suppose if a person comes up with something which you are not expecting at all, then you just sit there and think ‘oops, what am I supposed to say?’ […] You do feel vulnerable and in order to approach a question for mental health determining whether your patients are mild or moderate or severely depressed, you need to have that much confidence to remove your vulnerability. (Study 1, Group 2, Practice Nurse).

However, when nurses felt confident in dealing with mental health, normally through some previous experience or training in mental health, they viewed themselves as being able to take an holistic approach, which included encouraging discussion of mood. They were also more able to see a role for themselves (alongside the GP) in responding to patients.

I’ve got him coming back in six months time; he didn’t want to see anybody, but I thought it was planting the seeds to… you know, if he went home and thought about it and thought ‘well, actually maybe I do need to speak to somebody’ then he could come back and do that either at the [nurse led] clinic or with the GP. (Study 1, Group 3, Practice Nurse)

## Discussion

### Summary of main findings

This study has systematically examined both nurse (including specialist nurses) and GP perceptions of implementing case finding/screening for depression in DM and CHD patients using structured screening tools. Nurses’ views are particularly important given that this task is predominantly undertaken by practice nurses. We identified several features of the screening which may cause systematic under-detection of depression: namely difficulty incorporating screening into review consultations (including time pressures); replacing individualised with mechanistic assessment; a disconnection for nurses between physical and mental health, and uncertainties about care provision. Far from being a standardised process that encouraged detection of depression, we found participants describing screening as being conducted in a way which increases the chances of negative responses, and as an uncomfortable task when they lacked additional skills to provide immediate support to patients at the time of disclosure.

### Strengths and limitations

Participants in this study were self selecting and may therefore represent a set of professionals with research interests or particular interests in depression and depression management. Study 1 participants were recruited to a pilot trial of a nurse led intervention for depression in people with long term conditions (which happened to include QOF case finding as a method of identifying potential patient recruits) and Study 2 participants were recruited to a quality improvement project which included improving early detection. Therefore, their participation is unlikely to have been influenced by wanting to share their concerns about depression screening per se. Both studies recruited a mix of primary care practices, reflecting the general demographic mix of practice populations across Scotland. The focus groups were conducted for different project aims. However, both studies included questions aimed at eliciting views on how depression screening was conducted in everyday practice. The consistency of findings across two independent studies adds to the strength of their validity.

Some of the focus groups in Study 1 involve small numbers of participants and may be better described as joint interviews. Nonetheless, these often ensured that all participants were able to input their views. There may also be limitations in focus groups where participants come from a single general practice and therefore the group dynamic is affected in a way which limits voicing of different opinions, particularly where the power dynamic in a group is unbalanced. Conducting mixed groups including GPs and practice nurses from the same practice can include such a dynamic. However, the practical aspects of running focus groups in primary care needed to be acknowledged (practices normally only offered one day and time that would be suitable for the majority, and most were unable to travel to another surgery during the day or in the evening). Despite all efforts, expected participant numbers were often reduced on the day as other commitments took priority. However, both GPs and nurses appeared open in their self criticism and the limitations they perceived. Two group facilitators in each focus group probed for alternative opinions and ensured that all participants were included in discussion.

### Comparison with other studies

Two studies have shown case finding leads to improved depression outcomes in DM, but assessment of depression was carried out separately from the consultation (using diagnostic instruments) rather than within it [29,30]. Evidence from populations without chronic illness suggests that screening for depression in routine primary care is not effective in improving patient outcomes [11].

One reason for the poor impact of screening may simply be that those delivering the screening, such as nurses in the UK, have not been adequately trained. Taylor et al. found that variation in depression detection rates was related to experience and training in screening procedures [31]. A UK study of practice nurses found that 82% felt they lacked adequate knowledge and training in dealing with depression. Only a quarter had attended post-qualification mental health training and nurses rated mental health training a lower priority than physical illness in which they had already received more training [32]. The experience of the nurses in our study was one of being given a new task with minimal training. Therefore, the ‘initiation, enrolment and legitimation’ work required for cognitive participation are clearly lacking within the process of transferring this task to practice nurses.

Participants also reported other reasons why screening may be ineffective, in keeping with other studies [17,18]. Patients with depression work to maintain “face” in front of others [33] and both practitioners and patients are inclined to normalise distress in the face of the losses that come with a long term illness meaning that the possibility of depressive disorder may be missed or avoided [34]. Drawing on Normalization Process Theory, particularly as applied by Gunn to the implementation of depression care, it is clear there can be a lack of coherence by nurses in understanding depression (and who is, or is not depressed) that is not addressed within the delivery of routinized screening.

The cognitive participation of frontline staff is impacted by this lack of coherence, but more importantly, the use of screening tools seems to reduce opportunities for cognitive participation. The studies by Dowrick et al. and Leydon et al., found that doctors were cautious about the validity and utility of depression tools incentivised within the QOF, and considered their clinical judgement to be more important [17,18]. Our study findings more strongly indicate that the use of screening tools is viewed as actively restricting the use of clinical judgement and communication skills. Only Mitchell at al have previously included nurse perspectives on depression screening within chronic disease management reviews and they also reported a perceived lack of training, nurse discomfort in asking about mental health, and burden of screening, resulting in screening questions being avoided or not asked in full [19]. There is a clear lack of ‘legitimation’ on the part of nurses in relation to depression work that acts to prevent adoption of screening practices.

Our findings of implementation barriers, including replacing human interaction with mechanistic processes, the disconnect between physical and mental health, and uncertainties about care provision suggest that there are current deficits within the implementation of depression screening which limit the development of coherence and cognitive participation in depression care, particularly for primary care nurses.

### Implications for practice and policy and research

As several commentators have discussed, there can be negative consequences associated with incentivised activity [35,36] and possibly by formalising depression screening without adequate skills, an attempt to improve overall care may actually reduce holistic care. Simultaneously, it is important not to ‘throw the baby out with the bath water’ as there is a danger that the limitations of screening instruments or their poor application in practice might signal the end of case finding in these ‘at risk’ populations.

This research has identified lack of experience, lack of confidence and lack of time in health checks where screening is conducted as the key reasons why primary care nurses are reluctant to engage with mental health issues. Given that practice and specialist nurses’ current role in depression screening is significant, and that this may be extended to other primary healthcare roles (such as medical assistants), it is important that mental health awareness training is made more widely available to improve knowledge and confidence. This might include role play in how to enquire about mental health problems in patients. It could also be supported by mentoring/peer support by mental health nurses or primary care nurses with more experience in mental health.

The current default for nurses is not to engage in helping patients manage often minor mental health problems but to refer the patient to the GP. This may inhibit the potential for roll-out of self-help treatments within the primary care setting, which is a key goal for enhancing self care and for shifting the balance of care within long term condition management [37]. Additional support of the nurses by GPs until they become more comfortable with this work, and clear protocols for dealing with cases or concerns, including protocols for referral to the GP with indicative timescales (e.g. concern about someone who might be very depressed or suicidal) would also help to develop the relational work required for the adoption of screening practices. This support from GPs could also extend to providing and legitimising more time for nurse led health checks, even at the level of a ‘catch up’ slot at intervals during clinics.

## Conclusion

Primary care nurses implemented screening for depression in patients with chronic illness in ways which contribute to explaining the apparent small impact of screening. If screening is to be effective, barriers of time and resources, the dissonance between mechanistic and individualised aspects of care, and the disconnect between physical and mental health, must all be overcome. If nurses are to continue screening for depression there needs to be a more collective approach in primary care to enable its effective adoption which includes establishing support, training and systems and protocols that will ensure cognitive participation in depression work.

## Endnotes

^a^Whooley Questions:

• during the last month, have you often been bothered by feeling down, depressed or hopeless?; and

• during the last month, have you often been bothered by having little interest or pleasure in doing things?

^b^There are 40 Community Health Partnerships (CHPs) in Scotland, covering 14 Health Boards and 32 councils. A CHP is a committee of the Health Board which develops local community health services, in partnership with their local authority partners ensuring that health and social care services are integrated and seamless.

## Abbreviations

DM: Diabetes Mellitus; CHD: Coronary Heart Disease; NHS: National Health Service; QOF: Quality and Outcomes Framework; CHPs: Community Health Partnerships; PHQ-9: Patient Health Questionnaire; HADS: Hospital Anxiety and Depression Scale; BDI: Beck Depression Inventory II

## Competing interests

In both Study 1 and Study 2 all researchers were independent from the funding body, receiving no personal financial gain from the funders beyond re-imbursement to employing institutions for time spent on research activities.

Declaration of interests

M Maxwell declares there are no potential conflicts of interest

F Harris declares there are no potential conflicts of interest

C Hibberd declares there are no potential conflicts of interest

E Donaghy declares there are no potential conflicts of interest

R Pratt declares there are no potential conflicts of interest

C Williams declares there are no potential conflicts of interest

J Morrison declares there are no potential conflicts of interest

J Gibb declares there are no potential conflicts of interest

PWB Watson declares there are no potential conflicts of interest

C Burton declares there are no potential conflicts of interest.

## Authors’ contributions

MM wrote and revised this paper and contributed to discussions of key analytical themes across both studies. MM was PI in Study 1 and Research Lead for Study 2. RP was involved in analysis of findings in both studies and commented on drafts of this paper. CH, ED, FH and JG were responsible for data collection, with CH, ED and FH conducting coding and analysis. All commented on drafts of this paper. CB contributed to construction of analytical themes and to the writing of this paper. JM, CW, PW contributed to study design, discussion of findings and commented on drafts of the paper. *Responsibility for integrity and accuracy of data and analysis.* All contributing authors to this paper had access to all relevant papers (Information sheets, letters of introduction to study, focus group topic guides) and data necessary for verifying the integrity of the data and the accuracy of the analysis. Since these data combine data from two separate studies, care had to be taken that only anonymised data was shared across studies and only data relevant to the focus groups was shared. All authors read and approved the final manuscript.

## Pre-publication history

The pre-publication history for this paper can be accessed here:

http://www.biomedcentral.com/1471-2296/14/46/prepub
